# Music during anaerobic exercise in physically active adults: task-dependent evidence for repetition performance and affective valence, with uncertain maximal-performance effects

**DOI:** 10.3389/fspor.2026.1849596

**Published:** 2026-07-08

**Authors:** Derun Qiu, Xiao Teng, Zhiyi Chen, Liu Yang, Bo Zhao, Wenyuan Li

**Affiliations:** 1 College of P.E. and Sport, Beijing Normal University, Beijing, China; 2 Department of Radiology, The First Affiliated Hospital of Guangzhou University of Chinese Medicine, Guangzhou, China

**Keywords:** affective response, anaerobic exercise, meta-analysis, music, perceived exertion, performance, physically active adults, systematic review

## Abstract

**Background:**

Music is widely used during demanding exercise; however, its acute effects during anaerobic or predominantly high-intensity exercise remain uncertain, particularly when crossover designs and multiple task-specific effect sizes are considered.

**Objective:**

This study aims to evaluate the acute effects of music during anaerobic exercise in physically active adults using analyses that accounted for dependent effect sizes, crossover variance assumptions, task heterogeneity, risk of bias, and evidence certainty.

**Methods:**

Eligible effects were assessed at the effect-size level and synthesized using three-level random-effects models with cluster-robust variance estimation (CR2) by study. Sensitivity analyses included one-effect-size-per-study models, plausible within-person correlations for crossover studies, and exclusion of high-risk-of-bias studies where estimable.

**Results:**

The primary dataset included 113 effect sizes from 21 studies. Robust estimates showed uncertain effects for peak power, mean power, velocity, time-based performance, fatigue index, rating of perceived exertion, motivation, and enjoyment. Music was associated with higher repetitions [standardized mean difference = 0.46, 95% confidence interval (CI) 0.02–0.91] and improved feeling scale scores [mean difference (MD) = 0.75, 95% CI 0.35–1.15]. Heart rate was higher in the full robust analysis (MD = 5.33 bpm, 95% CI 1.91–8.75) but became uncertain after excluding high-risk-of-bias studies. Certainty was low or very low across outcomes.

**Conclusions:**

After accounting for dependence, task heterogeneity, and risk of bias, music during anaerobic exercise appears to be most plausibly linked to affective valence and selected repetition-based outcomes, whereas evidence for maximal performance enhancement remains limited and uncertain.

**Systematic Review Registration:**

https://www.crd.york.ac.uk/PROSPERO/view/CRD420261336754, identifier CRD420261336754.

## 1 Introduction

Music is commonly integrated into training and sport, but its practical value depends on the exercise context and the outcome under consideration. Across exercise and sport settings, music has been most consistently associated with changes in affective responses, motivation, and perceived exertion, whereas its effects on performance appear to be more context-dependent (1–3).

However, evidence specific to anaerobic or predominantly high-intensity exercise remains inconsistent. Some studies have reported favorable effects of music on exercise tolerance, repetition performance, motivation, or enjoyment, whereas others have shown little or no benefit for peak power, mean power, or related anaerobic indices (4–7). This inconsistency is unsurprising given the diversity of exercise models represented in the literature, including Wingate-type cycling tests, repeated-sprint protocols, sprint interval training, isometric tasks, resistance-based exercise, and sport-specific high-intensity efforts.

Mechanistically, music may plausibly influence exercise through attentional, affective, and motivational pathways; however, during very high-intensity or short-duration anaerobic tasks, rapidly accumulating physiological strain may reduce the extent to which external auditory stimuli can modify maximal mechanical output (1, 2, 8). This raises the possibility that music may show more reproducible effects on how exercise is experienced, such as perceived exertion, enjoyment, or affective valence, than on maximal performance indices themselves. Existing evidence syntheses provide useful background, whereas previous reviews have tended to focus more narrowly on specific task types, particularly the Wingate Anaerobic Test, rather than integrating performance, fatigue-related, affective, and physiological outcomes across the broader anaerobic domain (9).

This issue is particularly relevant for physically active adults, who commonly use music in real-world training settings and for whom both performance and exercise experience may be meaningful. Clarifying whether music meaningfully affects anaerobic exercise in this population could help refine both the interpretation of the current literature and the practical use of music as a low-cost adjunct in training.

Accordingly, this systematic review and meta-analysis aims to evaluate the acute effects of listening to music during anaerobic exercise on performance, fatigue-related outcomes, affective responses, and selected physiological responses in physically active adults. A secondary aim was to examine whether the evidence pattern differed by task family and music-intervention characteristics where sufficient data were available. We hypothesized that music would show more consistent favorable effects on perceptual and affective outcomes than on maximal or time-constrained anaerobic performance outcomes.

## 2 Methods

### 2.1 Protocol, registration, and reporting standard

This systematic review and meta-analysis was conducted in accordance with the Preferred Reporting Items for Systematic Reviews and Meta-Analyses (PRISMA 2020) statement and its updated guidance (10, 11). The protocol was prospectively registered in PROSPERO (CRD420261336754), and the review question and eligibility criteria followed the registered protocol. Analytical methods are described in the following.

### 2.2 Eligibility criteria

Eligibility criteria were defined *a priori* according to the participants, interventions, comparators, outcomes, and study design (PICOS) framework.

#### 2.2.1 Participants

Studies were eligible if they included adults aged 18 years or older who could reasonably be classified as physically active. Participants were considered physically active when the original report explicitly described them as physically active, recreationally active, trained, sport-participating, or regularly engaged in exercise or sport. Studies were excluded if regular exercise participation could not be judged from the report. Clinical populations, injured participants, and other special populations were excluded. Studies were also excluded if participants were explicitly described as having professional music or dance training.

#### 2.2.2 Intervention

The intervention of interest was listening to music during anaerobic exercise, such that music exposure and exercise occurred simultaneously. For the purposes of this review, eligible exercise tasks included anaerobic or predominantly anaerobic/high-intensity exercise models retained after screening. Studies were eligible regardless of music genre, tempo, or selection method, provided that music was delivered during the exercise task itself. Studies in which music was delivered only before exercise, only during recovery, or together with non-separable co-interventions were excluded.

#### 2.2.3 Comparator

Eligible studies were required to include a no-music control condition. Studies comparing different music conditions without a no-music comparator were excluded from the primary synthesis.

#### 2.2.4 Outcomes

Studies were eligible if they reported at least one outcome relevant to exercise performance, fatigue-related responses, affective responses, or related physiological/perceptual indicators. Primary outcome domains were performance outcomes (peak power, mean power, repetitions, velocity, time, and related objective indicators of anaerobic performance), fatigue-related outcomes [fatigue index and rating of perceived exertion (RPE)], and affective outcomes (feeling scale, motivation, enjoyment score, and related affective responses). Secondary outcomes included physiological responses such as heart rate.

#### 2.2.5 Study design

Only empirical experimental studies were eligible, including randomized or non-randomized controlled trials, crossover trials, and repeated-measures designs with a no-music control condition. Observational studies, reviews, conference abstracts, editorials, dissertations, and unpublished reports were excluded.

### 2.3 Information sources and search strategy

Electronic searches were conducted in PubMed, Web of Science, ScienceDirect, Scopus, and EBSCOhost (SPORTDiscus) from database inception to 10 March 2026. The reference lists of included studies and relevant reviews were also manually screened to identify additional eligible records. The complete database-specific search strings are provided in the search-strategy supplement.

Searches combined music- and exercise-related terms, and eligibility screening identified records involving anaerobic or predominantly high-intensity exercise. Searches were limited to English-language records.

### 2.4 Study selection

Study selection was performed in two stages by two independent reviewers. First, titles and abstracts were screened against the predefined eligibility criteria. Second, the full texts of potentially eligible studies were retrieved and assessed for final inclusion. Disagreements were resolved through discussion and, when required, adjudication by a third reviewer. The screening process is summarized in the PRISMA flow diagram, and full-text exclusion reasons are reported in the exclusion table given in the Supplementary Materials.

### 2.5 Data extraction

A standardized extraction form was used to capture study characteristics, participant characteristics, exercise protocol features, music-intervention characteristics, comparator details, outcome measures, measurement time points, and the numerical data required for quantitative synthesis. When outcome data were presented graphically, values were extracted using WebPlotDigitizer. The effect-size dataset included study and effect identifiers (study_id and effect_id), outcome, effect metric, design, task category, music selection, preference, tempo, timing, comparator, reporting quality, and risk-of-bias fields, where available.

Music interventions were coded using a standardized framework including selection method, preference, tempo or tempo category, genre, volume, familiarity, delivery timing, delivery mode, synchronous vs. asynchronous use, comparator, and reporting quality. Missing intervention features such as tempo, genre, volume, and delivery mode were coded as not reported unless explicitly described; synchronous vs. asynchronous use was classified from the protocol description. Reporting quality informed interpretation, and poorly reported studies were not automatically excluded from the primary synthesis. Details on screening reproducibility, extraction and coding, risk-of-bias judgments, effect-size calculation decisions, sensitivity analyses, and GRADE assessments are provided in Supplementary Tables S0–S16.

To minimize unit-of-analysis problems, each extracted effect size was assessed for eligibility before synthesis. Extracted effects were excluded from the primary analysis when the intervention was not music during the exercise task, when music was combined with a non-separable co-intervention, when the task was outside the acute anaerobic/high-intensity question, when the time point represented a pre-exercise or baseline measure, or when the outcome construct was not appropriate for the target outcome. Multiple eligible task-specific, set-specific, or load-specific effect sizes from the same study were retained only when they represented eligible primary contrasts and were subsequently handled statistically as dependent effects.

When studies included multiple eligible music arms sharing the same no-music comparator, the primary analysis prioritized the contrast most directly aligned with the review question, usually self-selected or preferred music during exercise vs. no music. Other eligible music arms, such as non-preferred, genre-specific, or tempo-manipulated conditions, were retained for secondary or sensitivity interpretation, where appropriate.

### 2.6 Risk-of-bias assessment

Risk of bias was assessed independently by two reviewers using RoB 2, the revised Cochrane risk-of-bias tool (12). Judgments were made for the randomization process, deviations from intended interventions, missing outcome data, measurement of the outcome, and selection of the reported result. Disagreements were resolved through discussion and, where necessary, consultation with a third reviewer.

### 2.7 Outcome classification

Outcomes were grouped *a priori* into performance outcomes (peak power, mean power, repetitions, velocity, time, and related objective indicators of anaerobic performance), fatigue-related outcomes (fatigue index, RPE, and related exertional measures), affective outcomes (feeling scale, motivation, enjoyment score, and related affective responses), and physiological outcomes (heart rate and other physiological indicators, where reported). Distinct constructs were not pooled solely because they were subjective; for example, discomfort ratings were not combined with RPE.

Exercise tasks were also coded at the effect-size level because anaerobic exercise is not a homogeneous exposure. The task categories were maximal_short_duration, repetition_resistance, repeated_effort_interval, isometric_endurance_like, and unclear. These categories were used to guide subgroup feasibility checks and the interpretation of task-dependent effects.

### 2.8 Data synthesis and statistical analysis

Meta-analysis was performed when at least two studies reported sufficiently comparable constructs within the same outcome domain and provided adequate numerical data. For outcomes measured on the same scale, effects were expressed as mean differences (MDs). For conceptually similar constructs measured using different scales, effects were expressed as standardized mean differences (SMDs). Positive values indicated higher values in the music condition, except for outcomes where lower values are inherently favorable, such as RPE and fatigue index; directionality was interpreted by outcome.

The primary analysis was performed in R using the metafor and clubSandwich packages. The main model was a three-level random-effects model fitted by restricted maximum likelihood, with effect sizes nested within studies. In metafor notation, the random-effects structure was specified as random = ∼1 | study_id/effect_id. Statistical inference used CR2 cluster-robust variance estimation, with study as the clustering unit.

Most included studies used crossover or repeated-measures designs, but within-person correlations and paired summary statistics were rarely reported. The primary analysis therefore used a plausible within-person correlation of *r* = 0.50 for crossover variance estimation when paired information was unavailable. Sensitivity analyses repeated the models using *r* = 0.30, 0.70, and 0.90 to examine the influence of this assumption and to avoid treating crossover data as independent-group data.

Model comparison tables included conventional fixed- or random-effects models, three-level restricted maximum likelihood (REML) models, CR2 robust estimates, and one-effect-size-per-study sensitivity models for each outcome. The CR2 estimates were treated as primary inferential results because they better addressed within-study dependence and small-sample uncertainty.

### 2.9 Subgroup analysis

Task-category subgroup analyses used the predefined categories maximal_short_duration, repetition_resistance, repeated_effort_interval, isometric_endurance_like, and unclear. Formal subgroup interpretation was restricted to outcome-by-task cells with at least three independent studies; cells below this threshold were treated as exploratory or narrative.

### 2.10 Sensitivity analysis

Sensitivity analyses included one-effect-size-per-study models, crossover-correlation assumptions (*r* = 0.30, 0.50, 0.70, and 0.90), and comparisons of conventional, three-level, and CR2 robust estimates. Exclusion of high-risk-of-bias studies and reporting-quality sensitivity analyses were conducted where sufficient study-level data remained after coding. When too few independent studies remained, the sensitivity analysis was not performed.

### 2.11 Publication bias

Small-study effects were assessed with funnel plots and Egger-type regression only when at least 10 independent studies were available for an outcome. When fewer than 10 studies were available, Egger-type regression was not performed.

### 2.12 Certainty of evidence

The certainty of evidence for each pooled outcome was evaluated using the GRADE approach (13). The profile explicitly considered risk of bias, inconsistency, indirectness, imprecision, and publication bias or small-study effects. Downgrading reasons were recorded for each outcome, with particular attention to lack of low-risk studies, sparse evidence, clustered effect sizes, wide robust confidence intervals, and expectancy or measurement concerns for subjective outcomes.

## 3 Results

### 3.1 Study selection

**Figure 1:**
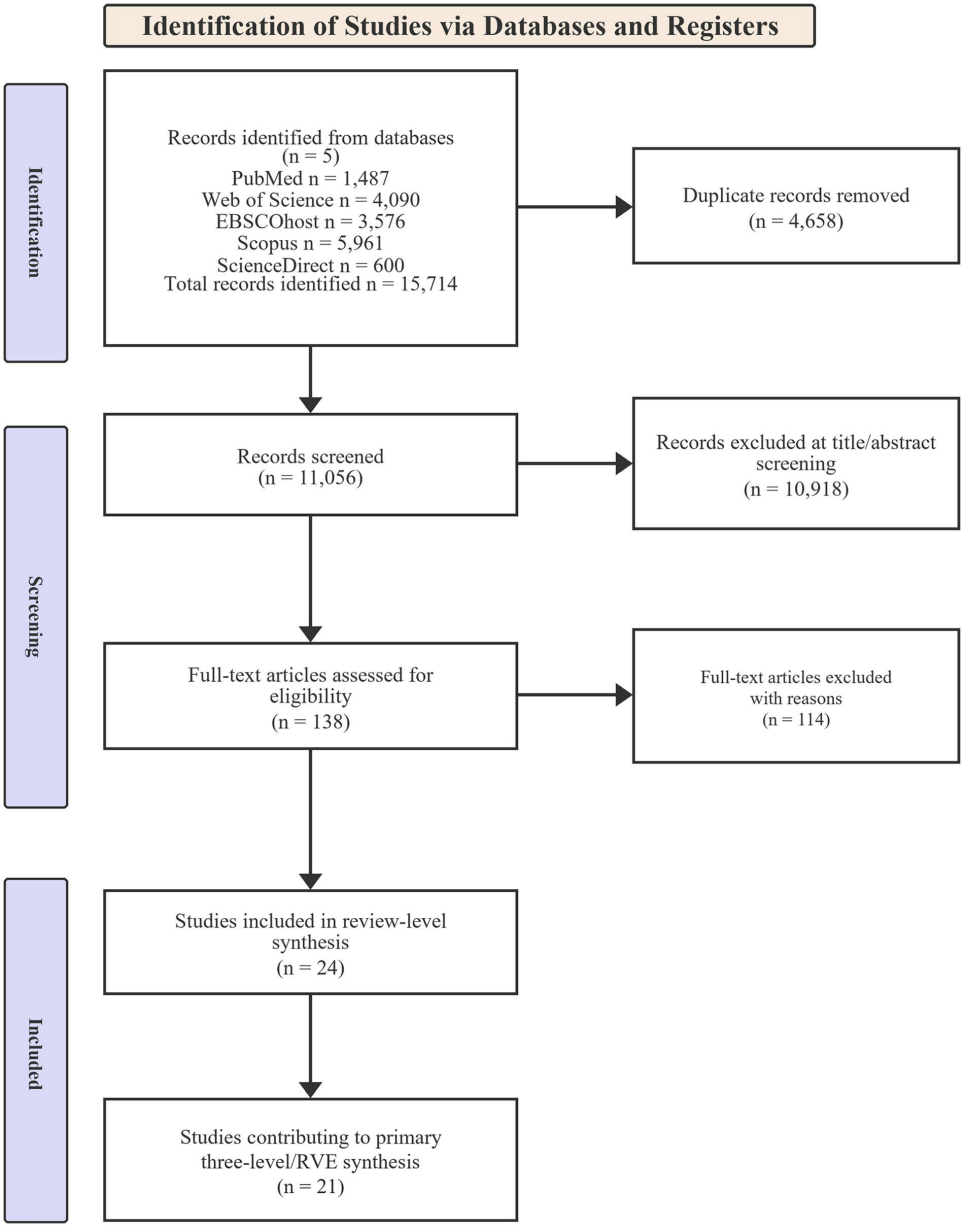
PRISMA flow diagram of study selection. The diagram shows the number of records identified, screened, assessed for eligibility, included in the review-level synthesis, and included in the primary three-level/robust variance estimation (RVE) synthesis.

The electronic search identified 15,714 records. After removal of 4,658 duplicates, 11,056 records remained for title and abstract screening. Of these, 10,918 records were excluded, leaving 138 full-text articles for eligibility assessment. Following full-text review, 114 articles were excluded, and 24 studies were included in the review. After effect-size-level eligibility assessment, 21 studies contributed at least one effect size to the primary robust synthesis. The study selection process is shown in Figure 1.

### 3.2 Study characteristics

The 24 included studies were published between 2012 and 2026 and predominantly enrolled physically active young adults. Most used crossover or repeated-measures experimental designs. Exercise tasks included Wingate-type tests, repeated-sprint protocols, sprint interval training, high-intensity interval exercise, isometric tasks, resistance exercise, and sport-specific high-intensity tasks. Music interventions varied substantially and included self-selected or preferred music, researcher-selected music, tempo-manipulated music, genre-specific music, and different timing strategies. Study characteristics are summarized in Table 1.

**Table 1:** Characteristics of included studies.

Study	*N*	Exercise task	Music/comparator conditions	Design	Outcomes assessed	Measurement time points
Kasai and Ando (14)	14	20-s maximal pedaling test	Control; unfamiliar-language music; self-selected familiar-language motivational music	Acute within-subject crossover	Relative peak power, mean power; motivation, arousal, pleasant emotion; prefrontal hemodynamics and autonomic activity	Rest; after music; after warm-up; after exercise
Cavaggioni et al. (15)	18	Running-based anaerobic sprint test (6-m × 35-m maximal sprints)	Preferred music; no music	Acute within-subject repeated measures	Maximum, mean, and minimum power; RPE; motivation	Post-test
Greco et al. (16)	26	Isometric leg-extension strength tests	Self-selected music; motivational music; control	Acute within-subject repeated measures	Peak force, rate of force development, mean force, fatigue index; FAS, feeling scale, RPE	Pre- and post-protocol
Miras-Moreno et al. (17)	15	Bench press, 4 sets at 70% 1-RM with 20% velocity loss	Control; knowledge of results; music; knowledge of results + music	Acute within-subject repeated measures	Repetitions; fastest and average velocity; felt arousal, feeling scale, RPE, RPD	Pre-session; after last set; across sets 1–4
Jebabli et al. (18)	19	2 sets of 5-m × 20-m repeated sprints	Preferred music during test; preferred music during warm-up; no music	Randomized crossover	Total sprint score, fast time index, fatigue index, pacing strategy; blood lactate, heart rate, RPE, feeling scale	Before, during, and after RSS test; set 1 and set 2
Poon et al. (19)	12	Plank-hold and wall-sit isometric tasks	Music during the entire exercise; music during fatigue only; no music	Randomized crossover	Time to fatigue perception, time to volitional exhaustion; heart rate; blood lactate	Before, at fatigue onset, and immediately after each task
Rogers et al. (20)	12	Countermovement jump and isometric mid-thigh pull	No music; non-preferred music; preferred music	Counterbalanced crossover	Peak force, rate of force development, jump height, peak power/velocity; motivation; feeling psyched up	During each test session; post-test
Jiménez-Roldán et al. (21)	14	Back squat velocity/power at 50% and 75% 1-RM; repetitions to failure at 75% 1-RM	Music; no music	Double-blind randomized crossover	Peak and mean power; mean and peak velocity; repetitions to failure; motivation; RPE	During exercise; post-muscular endurance test
Jones et al. (22)	18	Cycling HIIT: 10 s × 60 s at 100% Wmax with 75 s recovery	Respite music; continuous music; no music	Repeated-measures crossover	Affective valence, enjoyment, remembered pleasure	During work and recovery bouts; post-task
da Silva Duarte et al. (23)	12	Kickboxing-specific HIIE: 3-min × 2-min rounds with 1 min recovery	Self-selected music; no music	Randomized counterbalanced crossover	Number of strikes; LILS psychological states; RPE	Pre- and post-HIIE; end of each round
Garner et al. (24)	20	Bench press to failure	No sound; self-selected music; movement sonification	Within-subject repeated measures	Repetition maximum; beta power spectral density; frontal alpha asymmetry	Across trial and at 0%–100% elapsed time
Allocca Filho et al. (25)	11	Body-weight HIIT: 20-s × 30-s all-out/30-s recovery	Preferred music; non-preferred music; no music	Randomized repeated measures	Total movements, affective response, RPE, recovery, mood states; heart rate; lactate	Pre-, during, and post-session
Feiss et al. (26)	63	Wall-sit and plank-hold	No music; slow-tempo music; fast-tempo music	Randomized parallel groups with baseline and experimental trials	Time to exhaustion; RPE; attention allocation; pleasantness; arousal; heart rate	Baseline and experimental trial; every 30 s; task completion
Svoboda and Kostrna (27)	30	Isokinetic knee extension/flexion at 60°/s	Fast self-selected music; slow self-selected music; no music	Within-subject repeated measures	Peak extension/flexion torque; effort RPE; discomfort; attention; arousal; pleasantness	Post-exercise testing session
Silva et al. (28)	20	Handgrip strength and lat-pulldown strength-endurance tests	Preferred music genre; non-preferred music genre; no music	Randomized repeated measures	Maximal strength, strength-endurance; RPE	End of strength tests
Bozzato et al. (29)	20	Bench press power protocol: 3 sets of 8 repetitions at 75% 1-RM	Self-selected music; stimulative music; no music	Repeated-measures within-subject	Average and peak power; feeling scale; felt arousal; RPE	Pre-session; after warm-up; after training
D'Agata et al. (30)	17	Forearm plank to failure	Self-selected music; virtual reality; no external stimulus	Within-subject repeated measures	Plank duration; average heart rate	End of each trial
Atan (4)	28	RAST and Wingate anaerobic tests	Slow rhythm music; fast rhythm music; no music	Within-subject repeated measures	Peak and average power; fatigue index; heart rate; blood lactate	Rest and immediately post-test
Biagini et al. (31)	20	Bench press (3 sets to failure at 75% 1-RM) and squat jump	Self-selected music; no music	Counterbalanced crossover	Bench press repetitions; squat jump performance; takeoff velocity; RFD; POMS; RPE	Pre- and post-exercise; after sets/repetitions
Karageorghis et al. (32)	12	Track-based 400 m sprint/speed-endurance training	Synchronous music-based training; conventional training	Parallel-group longitudinal intervention	400-m performance; feeling scale; PACES; RPE; music liking	After each repetition/session and each time trial
Moss et al. (33)	16	Jump squat and bench throw at 30% 1-RM; back squat and bench press to failure at 60–80% 1-RM	No music; self-selected music; electronic dance music; metal	Repeated-measures within-subject	Peak/mean power; peak/mean velocity; repetitions to failure; heart rate; RPE; blood lactate; mood	Pre- and post-session; after every set
Patania et al. (34)	19	Walking at 6.5 km/h and 1-RM leg press	No music; low-, medium-, and high-tempo music	Within-subject repeated measures	Average and peak heart rate; walking RPE; leg press 1-RM; leg press RPE	During/end of walking; immediately after leg press
Stork and Martin Ginis (7)	20	Sprint interval training: 4 × 30 s Wingate bouts	Music; no music	Randomized counterbalanced crossover	Enjoyment, attitudes, intentions; RPE	Baseline; 60 min post-exercise; follow-up
Stork et al. (6)	20	Sprint interval training: 4 × 30 s Wingate bouts	Self-selected music; no music	Randomized counterbalanced crossover	Peak power; mean power; enjoyment; affect; task motivation; RPE	Pre-warm-up; each bout and rest; 30/60 min post-exercise

FAS, felt arousal scale; HIIT, high-intensity interval training; HIIE, high-intensity intermittent exercise; PACES, physical activity enjoyment scale; POMS, profile of mood states; RAST, running-based anaerobic sprint test; RPD, rating of perceived discomfort; RPE, rating of perceived exertion; RSS, repeated-sprint sets; Wmax, maximal aerobic power.

### 3.3 Risk of bias

**Figure 2:**
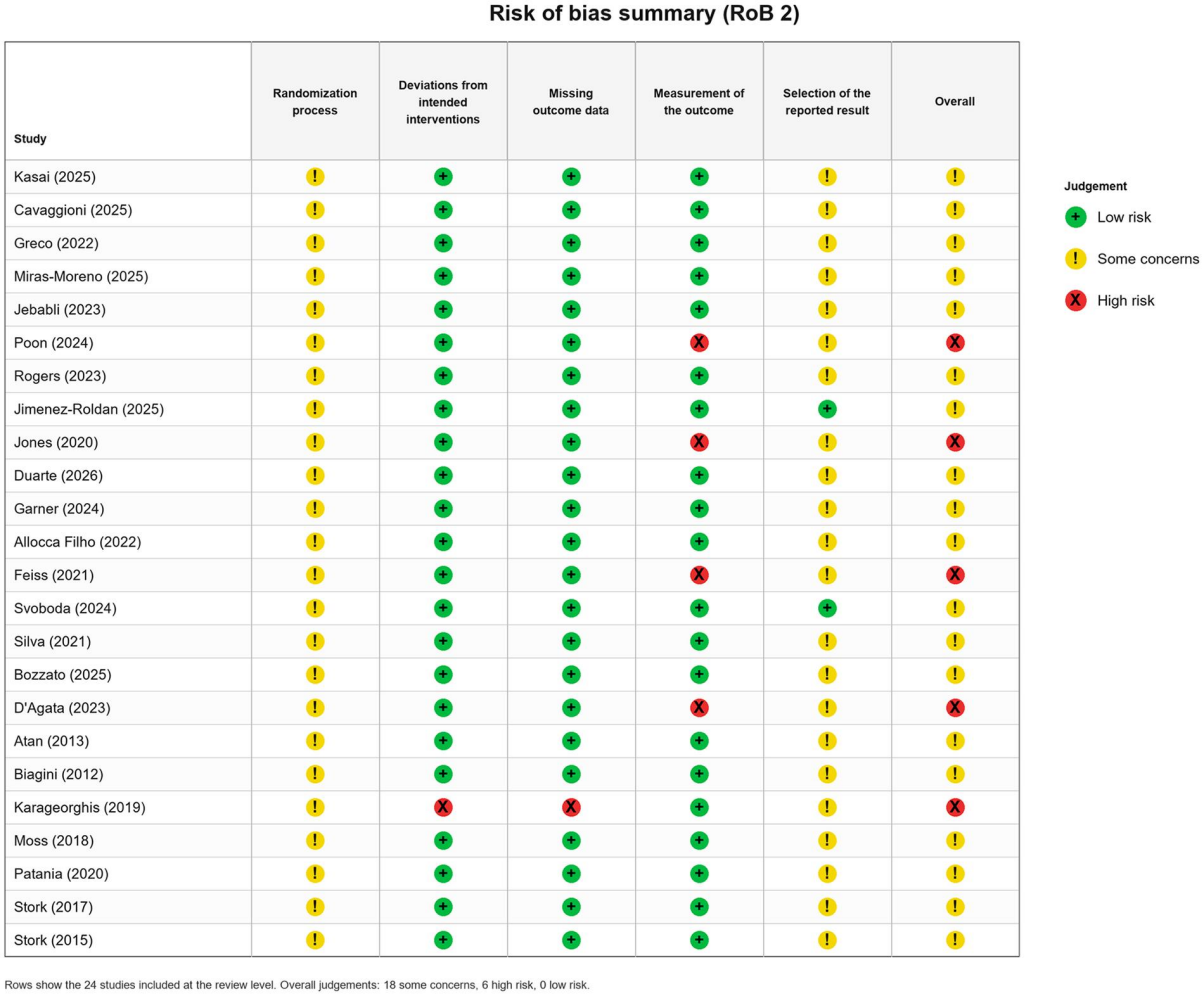
Risk-of-bias summary of included studies assessed using RoB 2. Judgments are presented by domain and overall risk-of-bias category.

No study was judged to be at low overall risk of bias. Eighteen studies were rated as having some concerns, and six were rated as high risk overall. Concerns most commonly arose from the randomization process and outcome measurement, whereas deviations from intended interventions and missing outcome data were generally less problematic. The overall risk-of-bias assessments are summarized in Figure 2.

### 3.4 Quantitative synthesis

After the effect-size-level eligibility assessment, 237 extracted effect sizes were eligible for detailed screening. Of these, 113 effect sizes from 21 studies were retained in the primary robust dataset, 61 were retained for secondary or sensitivity interpretation, and 63 were excluded from the primary synthesis because they did not match the acute music-during-exercise question, intervention contrast, time point, or outcome construct. Fifty-two effect sizes were retained for the one-effect-size-per-study sensitivity analyses.

**Figure 3:**
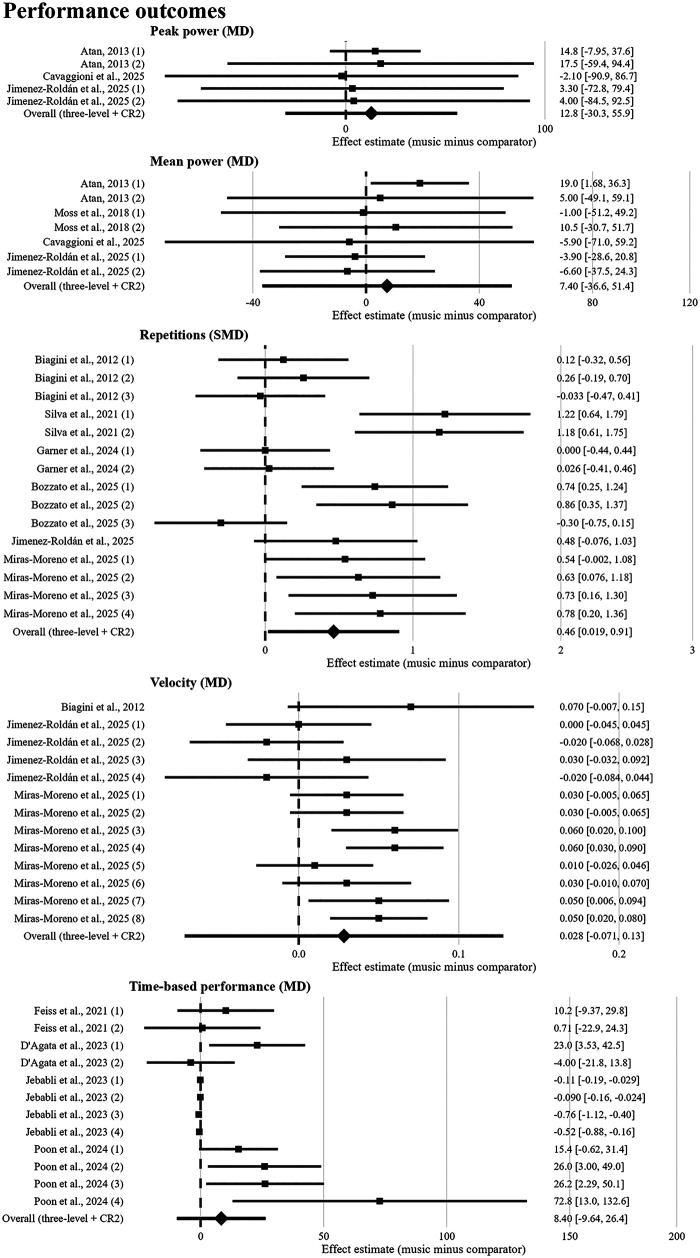
Forest plot for performance outcomes. Rows correspond to eligible effect sizes; repeated rows from the same study reflect dependent task, set, load, time point, or arm contrasts. Diamonds show three-level random-effects estimates with CR2 robust 95% confidence intervals. Values are music minus comparator.

**Figure 4:**
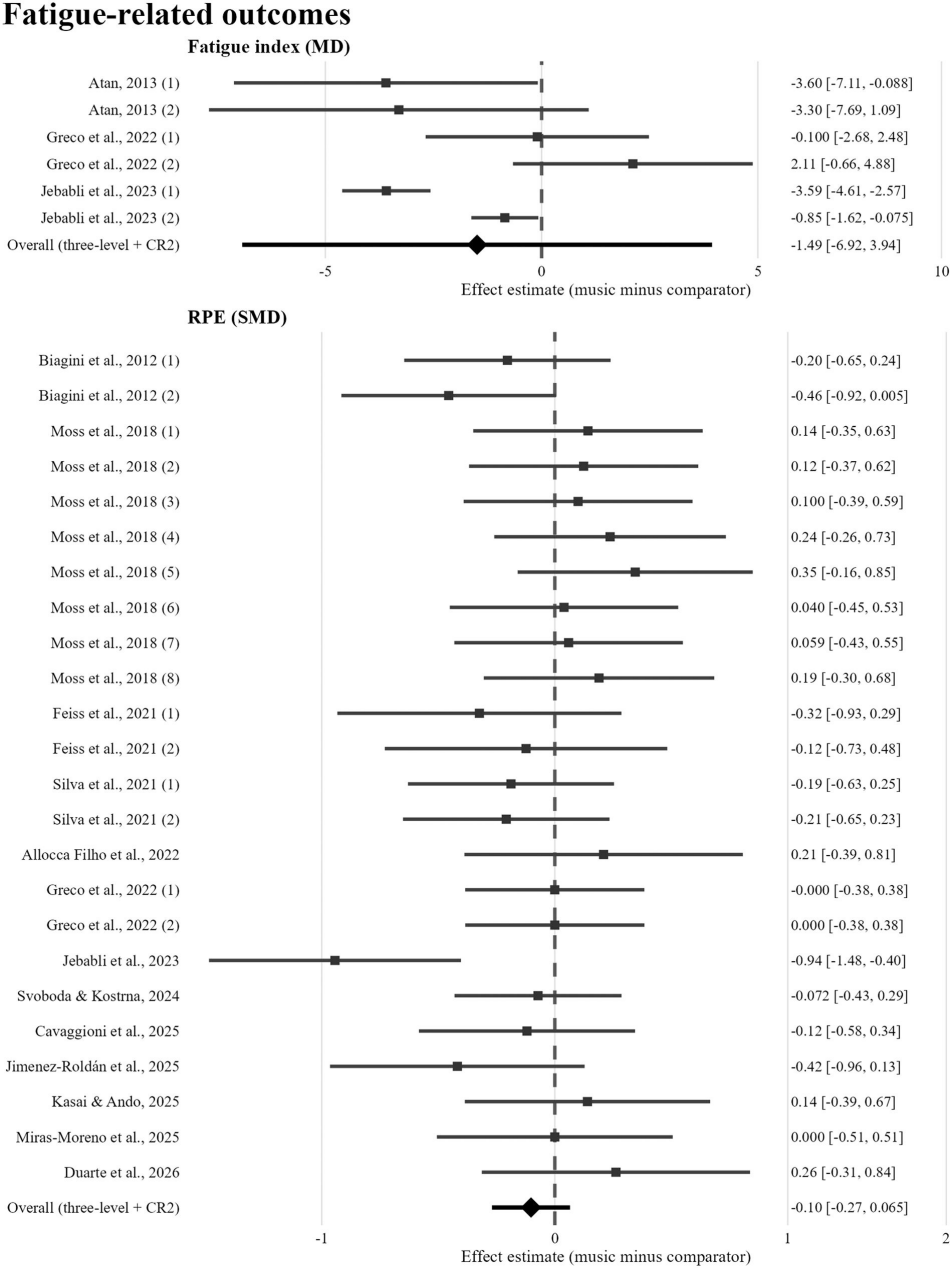
Forest plot for fatigue-related outcomes. Rows correspond to eligible effect sizes; repeated rows from the same study reflect dependent task, set, load, time point, or arm contrasts. Diamonds show three-level random-effects estimates with CR2 robust 95% confidence intervals. Values are music minus comparator.

**Figure 5:**
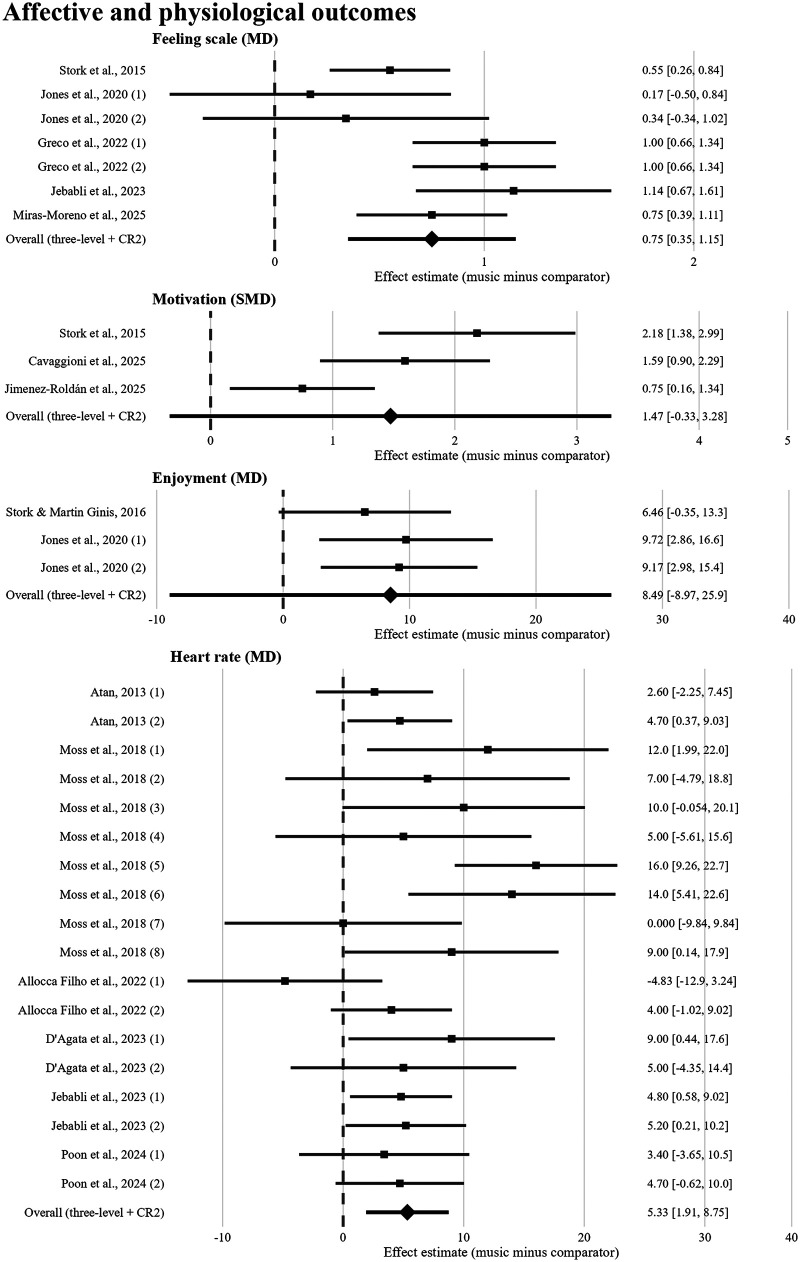
Forest plot for affective and physiological outcomes. Rows correspond to eligible effect sizes; repeated rows from the same study reflect dependent task, set, load, time point, or arm contrasts. Diamonds show three-level random-effects estimates with CR2 robust 95% confidence intervals. Values are music minus comparator.

The CR2 robust results were uncertain for most maximal or time-constrained performance outcomes. The most consistent favorable results were for repetition-based outcomes and the feeling scale. Heart rate was higher in the full robust analysis but became uncertain after high-risk-of-bias studies were excluded. RPE, motivation, and enjoyment were directionally favorable in some analyses but were not robust after accounting for dependence, risk of bias, and small-sample uncertainty. Forest plots for performance, fatigue-related, and affective/physiological outcomes are provided in Figures 3–5, respectively; pooled effects with certainty ratings are summarized in Table 2.

**Table 2:** Summary of pooled effects, robust inference, and certainty of evidence.

Outcome	Studies	Effect sizes	Effect (95% CI)	*P*-value	Certainty	Interpretation	Notes
Peak power	3	5	12.80 (−30.33 to 55.92)	0.196	Very low	The effect of music on peak power is very uncertain	CR2 robust
Mean power	4	7	7.40 (−36.63 to 51.44)	0.498	Very low	The effect of music on mean power is very uncertain	CR2 robust
Repetitions	6	15	0.46 (0.02 to 0.91)	0.044	Low	Low-certainty favorable signal for repetition-based outcomes	CR2 robust
Velocity	3	13	0.03 (−0.07 to 0.13)	0.298	Very low	The effect of music on movement velocity is very uncertain	CR2 robust
Time-based performance	4	12	8.40 (−9.64 to 26.44)	0.229	Very low	The effect of music on time-based performance is very uncertain	CR2 robust
Fatigue index	3	6	−1.49 (−6.92 to 3.94)	0.352	Very low	The effect of music on fatigue index is very uncertain	CR2 robust
Rating of perceived exertion	13	24	−0.10 (−0.27 to 0.07)	0.205	Low	Slightly lower but uncertain after robust inference	CR2 robust
Feeling scale	5	7	0.75 (0.35 to 1.15)	0.007	Low	Low-certainty favorable signal for affective valence	CR2 robust
Motivation	3	3	1.47 (−0.33 to 3.28)	0.072	Very low	Hypothesis-generating only	CR2 robust
Enjoyment	2	3	8.49 (−8.97 to 25.95)	0.102	Very low	The effect of music on enjoyment is very uncertain	CR2 robust
Heart rate	6	18	5.33 (1.91 to 8.75)	0.011	Very low	Higher in full analysis; uncertain after high-risk exclusion	CR2 robust

CI, confidence interval; GRADE, grading of recommendations assessment, development and evaluation; MD, mean difference; SMD, standardized mean difference. Certainty of evidence started at high because all pooled outcomes were derived from randomized or crossover intervention studies. Evidence was downgraded mainly for risk of bias, imprecision, and inconsistency. Publication bias was not used as a routine downgrading factor because most outcomes had too few independent studies for formal asymmetry testing.

### 3.5 Performance outcomes

#### 3.5.1 Peak power

Five effect sizes from three studies were retained in the primary robust analysis. The CR2 estimate did not show a clear difference between music and no-music conditions [MD = 12.80, 95% confidence interval (CI) −30.33 to 55.92, *p* = 0.196].

#### 3.5.2 Mean power

Seven effect sizes from four studies were retained. The robust estimate was uncertain and compatible with both lower and higher values under music conditions (MD = 7.40, 95% CI −36.63 to 51.44, *p* = 0.498).

#### 3.5.3 Repetitions

Fifteen effect sizes from six studies were retained for repetition or repetition-to-failure outcomes. The robust estimate favored music (SMD = 0.46, 95% CI 0.02–0.91, *p* = 0.044). The one-effect-size-per-study analysis was directionally consistent (SMD = 0.51, 95% CI 0.12–0.90, *p* = 0.010), but the evidence should be interpreted cautiously because the studies were few and the task contexts were heterogeneous.

#### 3.5.4 Velocity

Thirteen effect sizes from three studies were retained. Although conventional models suggested a small positive estimate, the three-level CR2 analysis did not show a robust effect (MD = 0.03, 95% CI −0.07 to 0.13, *p* = 0.298).

#### 3.5.5 Time-based performance

Twelve effect sizes from four studies were retained. The robust estimate was uncertain (MD = 8.40, 95% CI −9.64 to 26.44, *p* = 0.229), and the direction varied across task contexts and sensitivity assumptions.

### 3.6 Fatigue-related outcomes

#### 3.6.1 Fatigue index

Six effect sizes from three studies were retained. The CR2 estimate did not show a clear difference between music and no-music conditions (MD = −1.49, 95% CI −6.92 to 3.94, *p* = 0.352).

#### 3.6.2 Rating of perceived exertion

Twenty-four effect sizes from 13 studies were retained after excluding baseline measures, warm-up-only music contrasts, non-anaerobic tasks, and discomfort ratings that did not represent RPE. The robust estimate was directionally lower under music but was uncertain (SMD = −0.10, 95% CI −0.27 to 0.07, *p* = 0.205). Thus, the analysis did not support a strong conclusion that music reliably reduces RPE during anaerobic exercise.

### 3.7 Affective outcomes

#### 3.7.1 Feeling scale

Seven effect sizes from five studies were retained. The robust estimate was directionally favorable under music conditions (MD = 0.75, 95% CI 0.35–1.15, *p* = 0.007), consistent with a low-certainty signal for more positive affective valence during or after anaerobic exercise.

#### 3.7.2 Motivation

Three effect sizes from three studies were retained. The conventional and three-level REML estimates were positive, but the CR2 robust confidence interval was wide and crossed the null (SMD = 1.47, 95% CI −0.33 to 3.28, *p* = 0.072). This finding should therefore be treated as hypothesis-generating.

#### 3.7.3 Enjoyment score

Three effect sizes from two studies were retained. The CR2 robust estimate was imprecise and did not provide a stable basis for strong inference (MD = 8.49, 95% CI −8.97 to 25.95, *p* = 0.102).

### 3.8 Physiological outcomes

#### 3.8.1 Heart rate

Eighteen effect sizes from six studies were retained. Music was associated with higher heart rate values in the full robust analysis (MD = 5.33, 95% CI 1.91–8.75, *p* = 0.011). However, after excluding high-risk-of-bias studies, the estimate remained directionally higher but became uncertain (MD = 5.24, 95% CI −0.63 to 11.11, *p* = 0.066). This outcome should therefore be interpreted as a very-low-certainty signal of possible physiological activation or arousal rather than as evidence that music directly improves anaerobic performance.

### 3.9 Subgroup analysis

**Figure 6:**
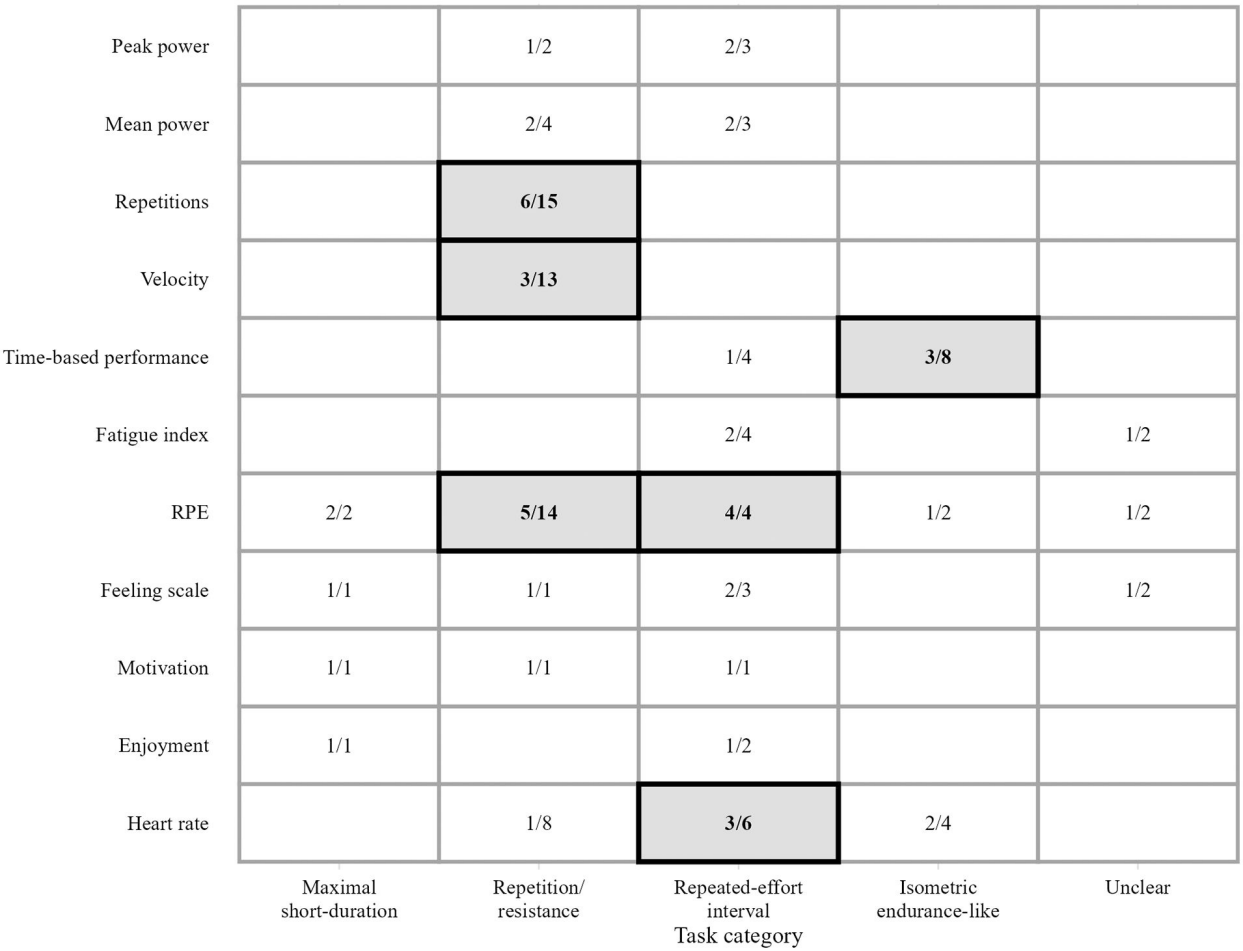
Task-category subgroup feasibility map. Cells show independent studies/effect sizes; outlined cells met the predefined threshold of at least three independent studies for formal subgroup interpretation.

Task-category analyses were feasible only for a limited set of outcome-by-task combinations. Combinations meeting the minimum threshold included heart rate: repeated_effort_interval (three studies); repetitions: repetition_resistance (six studies); rating of perceived exertion: repetition_resistance (five studies); rating of perceived exertion: repeated_effort_interval (four studies); time-based performance: isometric_endurance_like (three studies); and velocity: repetition_resistance (three studies). Other task and music-characteristic comparisons were too sparse for reliable formal subgroup testing and were therefore interpreted narratively. The feasibility pattern is summarized in Figure 6.

The task-category evidence was consistent with task-dependent effects rather than a single pooled anaerobic effect. Repetition outcomes were concentrated in repetition_resistance tasks, whereas peak and mean power were mostly derived from maximal_short_duration or repeated_effort_interval tasks. Preference and music-selection analyses for motivation, enjoyment, and affective valence were limited by the small number of independent studies and incomplete reporting of music characteristics.

### 3.10 Sensitivity analyses

The one-effect-size-per-study analyses were generally consistent with the primary interpretation. Repetitions remained directionally favorable (SMD = 0.51, 95% CI 0.12–0.90, *p* = 0.010), feeling scale scores remained favorable (MD = 0.76, 95% CI 0.49–1.03, *p* < 0.001), and heart rate remained higher under music conditions (MD = 3.46, 95% CI 0.93–5.98, *p* = 0.007). In contrast, peak power, mean power, velocity, time-based performance, fatigue index, and RPE remained uncertain or did not provide robust evidence of benefit.

Crossover-correlation sensitivity analyses using *r* = 0.30, 0.50, 0.70, and 0.90 showed that most outcome directions were not materially altered by the assumed within-person correlation. RPE remained uncertain across the correlation assumptions. For peak power, the estimate crossed the conventional *p* < 0.05 threshold only when *r* = 0.90 was assumed; because paired correlations were rarely reported, this isolated sensitivity result was interpreted cautiously and was not treated as robust evidence of benefit.

RoB 2 mapping identified no low-risk studies in the primary dataset and four high-risk studies contributing primary effect sizes. Excluding high-risk studies did not change the results for outcomes with no high-risk studies in the relevant synthesis. RPE remained uncertain (SMD = −0.09, 95% CI −0.28 to 0.09, *p* = 0.271), feeling scale scores remained favorable (MD = 0.84, 95% CI 0.42–1.26, *p* = 0.009), and heart rate became uncertain (MD = 5.24, 95% CI −0.63 to 11.11, *p* = 0.066). Time-based performance and enjoyment could not be robustly re-estimated after high-risk exclusion because fewer than two independent studies remained.

### 3.11 Publication bias

**Figure 7:**
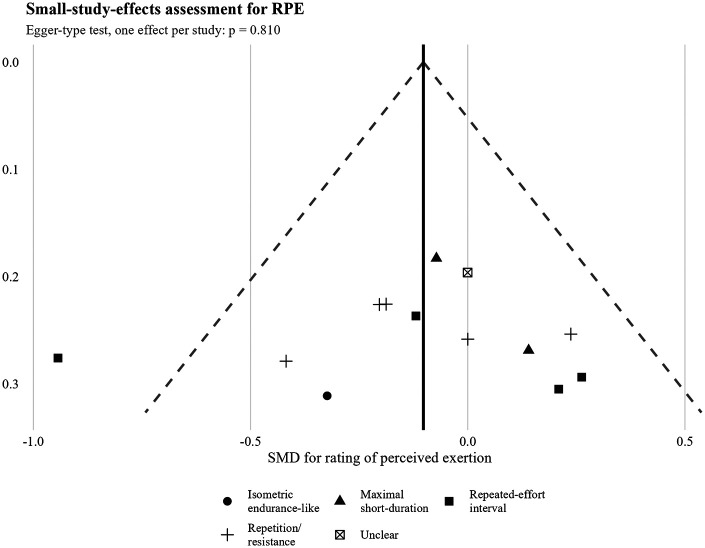
Funnel plot and small-study-effects assessment for rating of perceived exertion. One effect size per study is displayed; dashed lines indicate pseudo-95% limits around the RPE summary estimate. Egger-type regression did not indicate asymmetry (*p* = 0.810).

Only RPE had at least 10 independent studies and was therefore assessed for small-study effects. Egger-type regression was applied to the one-effect-size-per-study dataset to avoid dependence among effects from the same study. The test did not indicate asymmetry (*p* = 0.810), but the number of studies was modest, so the result should be interpreted cautiously (Figure 7). Egger-type tests were not conducted for other outcomes.

### 3.12 Certainty of evidence

Using the GRADE approach, the certainty of evidence was judged to be low or very low across outcomes. The most common reasons for downgrading were the absence of low-risk-of-bias studies, sparse evidence, clustered effect sizes, wide CR2 robust confidence intervals, sensitivity to high-risk exclusion, and expectancy or measurement concerns for subjective outcomes. Evidence was low for repetitions, RPE, and feeling scale and very low for peak power, mean power, velocity, time-based performance, fatigue index, motivation, enjoyment, and heart rate. Table 2 summarizes the pooled estimates, robust inference, and certainty ratings.

Taken together, the certainty ratings support cautious conclusions. The evidence for repetition performance and feeling scale scores was rated as low, and the evidence for other outcomes was rated as low or very low due to risk of bias, imprecision, inconsistency, indirectness, and dependence among multiple effect sizes from the same studies.

## 4 Discussion

### 4.1 Principal findings

The present review indicates that the acute effects of music during anaerobic exercise are task-dependent and less certain than suggested by conventional inverse-variance models alone. In three-level models with CR2 robust variance estimation, music was not clearly associated with improvements in peak power, mean power, movement velocity, time-based performance, fatigue index, or RPE. Low-certainty favorable estimates were observed for repetition-based outcomes and the feeling scale, whereas heart rate was higher only in the full robust analysis and became uncertain after excluding high-risk studies.

Overall, the findings do not support a uniform ergogenic effect of music across anaerobic exercise tasks. Music exposure was associated with more favorable affective valence and repetition-based outcomes in some contexts, but the evidence does not justify a broad claim that music reliably improves maximal or time-constrained anaerobic performance. Motivation and enjoyment were directionally favorable in conventional models, but the robust estimates were too imprecise to support strong conclusions.

### 4.2 Potential mechanisms and task-dependent interpretation

Music may influence anaerobic exercise through attentional, affective, and motivational pathways, but the included studies did not directly test these mechanisms. Mechanistic explanations are therefore hypotheses rather than demonstrated causal pathways.

Task structure may help explain the pattern of findings. Repetition-based tasks allow more time for music to influence effort tolerance, affective appraisal, and persistence, whereas peak power, mean power, and velocity are usually produced within brief maximal efforts.

Heart rate and intervention timing require similar caution. Higher heart rate should not be interpreted as a performance benefit, and effects were excluded when music was delivered only before exercise, combined with other stimuli, or linked to outcomes outside the review constructs. These issues support task-specific interpretation rather than a single pooled anaerobic effect across Wingate-type, repeated-sprint, resistance-repetition, and isometric endurance-like tasks. This task-specific interpretation is also consistent with prior evidence that music preference, auditory stimuli, and affective responses can influence exercise responses across resistance and continuous-exercise contexts (36–41).

### 4.3 Comparison with previous reviews and primary studies

The present findings are broadly consistent with previous evidence syntheses but are more task-specific. The closest prior review focused mainly on Wingate-derived outcomes (9), whereas this review included a broader range of anaerobic and predominantly high-intensity exercise models. This broader scope likely increased between-task variability, making a generalized ergogenic interpretation less tenable.

The low-certainty signals for feeling scale and repetition performance are also compatible with broader exercise-psychology evidence suggesting that music can influence affect, perceived effort, arousal, and persistence. In the present evidence base, however, these pathways were not consistently associated with performance improvements across all anaerobic tasks.

This task-specific interpretation helps explain why favorable findings in sprint interval or resistance studies can appear alongside null or mixed findings in Wingate-type or swimming-based protocols (5–7, 35).

### 4.4 Practical implications

In practice, music may be considered as an individualized aid for repetition-based or tolerance-oriented anaerobic tasks, especially when the aim is to support affect or persistence. The evidence is insufficient to recommend music as a general performance-enhancing strategy for maximal power or sprint-based tasks.

The evidence does not support the claim that music is a universally effective performance enhancer for anaerobic exercise. Practitioners should be especially cautious when expecting benefits for peak power, mean power, sprint or movement velocity, or time-based performance. Music selection may reasonably be individualized, but the current evidence is too sparse to identify a single optimal tempo, genre, preference strategy, or delivery mode across outcomes.

### 4.5 Strengths and limitations

Key strengths were prospective registration, duplicate screening and extraction, standardized task and music-intervention coding, and analyses that accounted for dependent effect sizes, crossover assumptions, risk of bias, and evidence certainty.

The main limitations were methodological. No included study was judged to be at low risk of bias, blinding was difficult for subjective outcomes, and many crossover studies did not report within-person correlations or paired summary statistics. Three-level modeling, CR2 robust inference, and correlation sensitivity analyses addressed these issues only partially.

Heterogeneity and reporting limitations were also important. Tasks, music characteristics, and outcomes varied, intervention reporting was often incomplete, and formal subgroup analyses were possible only for a few outcome-by-task combinations. Small-study-effects assessment was limited to RPE and should be interpreted cautiously.

### 4.6 Future research directions

Future trials should report music characteristics in detail, including tempo, genre, volume, familiarity, preference, timing, delivery mode, and comparator condition. They should also prespecify the target task and outcome construct, report sufficient data for paired or crossover analyses, and avoid combining music with other stimuli unless the independent effect of music can be estimated.

First, crossover trials should report the paired statistics needed for synthesis, including within-person correlations or change-score standard deviations. Second, trials should prespecify primary outcomes and distinguish repeated measurements within the same participants from independent comparisons. Third, intervention reporting should include selection method, preference, tempo, genre, familiarity, volume, delivery timing, delivery mode, and synchronous or asynchronous use.

Fourth, future research should examine task families separately, including maximal short-duration tasks, repetition-resistance tasks, repeated-effort interval protocols, and isometric endurance-like tasks. Finally, because the most plausible benefits appear to be related to affective valence and effort tolerance, future studies should test whether acute improvements translate into a willingness to repeat high-intensity exercise, training adherence, or longer-term behavior.

## 5 Conclusion

In physically active adults, music during anaerobic exercise appears to be a task-dependent and low-certainty intervention rather than a universally ergogenic aid. After accounting for dependent effect sizes, crossover variance assumptions, task heterogeneity, and risk of bias, evidence for improvements in peak power, mean power, velocity, time-based performance, fatigue index, RPE, motivation, and enjoyment was uncertain. Low-certainty favorable signals were observed for repetition-based outcomes and affective valence; however, these findings require confirmation in better-reported and adequately powered task-specific trials. Heart rate was higher in the full robust analysis but became uncertain after excluding high-risk studies, so it should not be interpreted as a direct performance benefit. Overall, the findings suggest a cautious, individualized approach to music during demanding exercise rather than broad performance claims.

## Data Availability

The original contributions presented in the study are included in the article/Supplementary Material; further inquiries can be directed to the corresponding author.
