# Supplementary material for: Music during anaerobic exercise in physically active adults: task-dependent evidence for repetition performance and affective valence, with uncertain maximal-performance effects
Source: Front Sports Act Living. 2026 Jul 8;8:1849596. doi: 10.3389/fspor.2026.1849596 (PMC13388559; doi:10.3389/fspor.2026.1849596)
Supplement: Supplementary file 1 [file Table1.docx]

| **Report Reference** | **Justification for exclusion** |
| --- | --- |
| Effects of rhythmical and extra-rhythmical qualities of music on heart rate during stationary bike activities | **WO** |
| Refining Music Tempo for an Ergogenic Effect on Stationary Cycling Exercise | **WI** |
| Does the Musical Tempo Enhance Physical Performance? | **WI** |
| Effects of Musical Tempo on Physiological, Affective, and Perceptual Variables and Performance of Self-Selected Walking Pace | **WI** |
| Exploring Music Intensity on Heart Rate, Perceived Exertion and Physical Performance During Sub-Maximal Exercise | **WL** |
| Effect of Music and Rhythm on Physical Performance | **WI** |
| A Series of Acute Psychological Priming Interventions Assessing Changes in Hormonal and Physical Performance Measures During Resistance Training | **WI** |
| Effects of Music on Work-Rate Distribution During a Cycling Time Trial | **WI** |
| Put Some Music on: The Effects of pre-Task Music Tempo on Arousal, Affective State, Perceived Exertion, and Anaerobic Performance | **WP** |
| The Effects of Mental Imagery and Music on Sprint Swimming Performance | **WP** |
| Effects of Listening to Preferred versus Non-Preferred Music on Repeated Wingate Anaerobic Test Performance | **WP** |
| Effects of Music During Exercise in Different Training Status | **WI** |
| Effects of Self-Selected or Randomly Selected Music on Performance and Psychological Responses During a Sprint Interval Training Session | **WP** |
| A Motivational Music and Video Intervention Improves High-Intensity Exercise Performance | **WI** |
| Effects of Music on Repeated-Sprint Performance of Elite Tunisian Soccer Players: Comparing Morning to Afternoon Practice Sessions | **WP** |
| One-HIIT Wonder: Can Music Make High-Intensity Interval Training More Pleasant? | **WI** |
| The Effects of Music on Exercise as Determined by Physiological Recovery Heart Rates and Distance | **WI** |
| Effect of Passive Distraction on Treadmill Exercise Test Performance in Healthy Males Using Music | **WI** |
| Influence of Music and Its Moments of Application on Performance and Psychophysiological Parameters During a 5 km Time Trial | **WL** |
| Cerebral Mechanisms Underlying the Effects of Music During a Fatiguing Isometric Ankle-Dorsiflexion Task | **WO** |
| How Does Music Aid 5 km of Running? | **WI** |
| Effects of Music and Music-Video on Core Affect During Exercise at the Lactate Threshold | **WI** |
| Preferred Music Genre Benefits During Strength Tests: Increased Maximal Strength and Strength-Endurance and Reduced Perceived Exertion | **WP** |
| Effects of Music, Video, and 360-Degree Video on Cycle Ergometer Exercise at the Ventilatory Threshold | **WI** |
| The Effect of Music Listening on Running Performance and Rating of Perceived Exertion of College Students | **WI** |
| The Effect of Subliminal Music on Muscle Tone, Pain, and Vital Signs in Female Athletes: A Community-Based Clinical Trial Based on the Schopenhauer’s Theory | **WP** |
| The Effects of Sensory Deprivation and Music on Perceived Exertion and Affect During Exercise | **WI** |
| When it HIITs You, You Feel No Pain: Psychological and Psychophysiological Effects of Respite–Active Music in High-Intensity Interval Training | **WD** |
| Difference In Wingate Power Output In Response To Music as Motivation | **WP** |
| Effects of Music on Physiological and Affective Responses to Graded Treadmill Exercise in Trained and Untrained Runners | **WI** |
| Spontaneous Velocity Effect of Musical Expression on Self-Paced Walking | **WI** |
| Optimizing Beat Synchronized Running to Music | **WI** |
| Effects of Types and Intensities of Background Music on Treadmill Endurance | **WI** |
| Tolerance to Exercise Intensity Modulates Pleasure When Exercising in Music: The Upsides of Acoustic Energy for High Tolerant Individuals | **WI** |
| Carry-Over Effects of Music in an Isometric Muscular Endurance Task | **WP** |
| Effects of Familiar and Unfamiliar Asynchronous Music on Treadmill Walking Endurance | **WI** |
| Effects of Music and Video on Perceived Exertion During High-Intensity Exercise | **WI** |
| Perceived Importance of Components of Asynchronous Music During Circuit Training | **WD** |
| Effects of Listening to Preferential Music on Sex Differences in Endurance Running Performance | **WI** |
| The Influence of Rhythm and Personality in the Endurance Response to Motivational Asynchronous Music | **WP** |
| Temporal Analysis of the Ergogenic Effect of Asynchronous Music on Exercise | **WL** |
| The Effects of Tai Chi Practice With Asynchronous Music on Compliance and Fall-Related Risk Factors in Middle-Aged and Older Women: A Pilot Study | **WP** |
| Effects of Music Tempo on Performance, Psychological, and Physiological Variables During 20 km Cycling in Well-Trained Cyclists | **WI** |
| The Effects of Music Preference and Exercise Intensity on Psychological Variables | **WO** |
| The Effects of Music Tempo and Loudness Level on Treadmill Exercise | **WI** |
| The Effect of Motivational Music on Sub-Maximal Exercise | **WI** |
| Listening to Motivational Music Mitigates Heat-Related Reductions in Exercise Performance | **WI** |
| The Effect of Motivational and Relaxation Music on Aerobic Performance, Rating Perceived Exertion and Salivary Cortisol in Athlete Males | **WI** |
| Listening to Motivational Music: Lactate and Cortisol Response to a Single Circuit Resistance Exercise for Young Male Athletes | **WP** |
| Impact Music on Some of Physical Readiness Factors in Exercised and Unexercised Girl Student | **WO** |
| The Effect of Music as a Motivational Tool on Isokinetic Concentric Performance in College Aged Students | **WP** |
| Perceived Exertion: Influence of Expected, Unexpected Relief and Frustration on Time to Exhaustion | **WO** |
| The Effect of Music on 10-km Cycle Time-Trial Performance | **WI** |
| Effects of Music on Mood During Bench Stepping Exercise | **WP** |
| The Influence of Self-Selected Music on Affect-Regulated Exercise Intensity and Remembered Pleasure During Treadmill Running | **WI** |
| Moderating Influence of Dominant Attentional Style and Exercise Intensity on Responses to Asynchronous Music | **WO** |
| See Hear: Psychological Effects of Music and Music-Video During Treadmill Running | **WI** |
| The Relationship Between Exercise Intensity and Preferred Music Intensity | **WO** |
| The Influence of Asynchronous Motivational Music on a Supramaximal Exercise Bout | **WP** |
| Listening to Preferred Music Improved Running Performance without Changing the Pacing Pattern during a 6 Minute Run Test with Young Male Adults | **WI** |
| Can High-Intensity Exercise Be More Pleasant? Attentional Dissociation Using Music and Video | **WI** |
| Interactive Effects of Visual and Auditory Intervention on Physical Performance and Perceived Effort | **WI** |
| Psychological Effects of Music Tempi during Exercise | **WI** |
| Psychological, Psychophysical, and Ergogenic Effects of Music in Swimming | **WI** |
| Psychophysical and Ergogenic Effects of Synchronous Music During Treadmill Walking | **WI** |
| Effect of Motivational Music on Psychological and Physiological Responses During High-Intensity Interval Training | **WO** |
| Effect of Different Music Tempos on Aerobic Performance and Recovery | **WI** |
| In Dubio Pro Silentio – Even Loud Music Does Not Facilitate Strenuous Ergometer Exercise | **WI** |
| Influence of Music on Maximal Self-Paced Running Performance and Passive Post-Exercise Recovery Rate | **WI** |
| Psychophysiological Effects of Synchronous versus Asynchronous Music During Cycling | **WI** |
| Listening to Music in the First, but not the Last 1.5 km of a 5-km Running Trial Alters Pacing Strategy and Improves Performance | **WI** |
| Effects of Psychological Priming, Video, and Music on Anaerobic Exercise Performance | **WI** |
| Influence of Music on Performance and Psychophysiological Responses During Moderate-Intensity Exercise Preceded by Fatigue | **WI** |
| Music and Physical Activity in Psychological Well-Being | **WO** |
| High Tempo Music Prolongs High Intensity Exercise | **WP** |
| Effects Music Has on Lap Pace, Heart Rate, and Perceived Exertion Rate During a 20-Minute Self-Paced Run | **WI** |
| The Effects of Music on the Perceived Exertion Rate and Performance of Trained and Untrained Individuals During Progressive Exercise | **WI** |
| The Perception of Exertion and Cardiorespiratory Responses of Rhythmic Exercise Performed to Music Compared with Treadmill Walking Under Three Different Tempos in Postmenopausal Women | **WP** |
| Effects of Preferred and Nonpreferred Music on Continuous Cycling Exercise Performance | **WI** |
| The Heat Is On: Effects of Synchronous Music on Psychophysiological Parameters and Running Performance in Hot and Humid Conditions | **WI** |
| Beneficial Effect of Preferential Music on Exercise Induced Changes in Heart Rate Variability | **WO** |
| Psychophysiological and Ergogenic Effects of Music in Swimming | **WI** |
| Influence of Music on Ratings of Perceived Exertion During 20 Minutes of Moderate Intensity Exercise | **WI** |
| Influence of Music on Wingate Anaerobic Test Performance | **WP** |
| The Effects of Listening to Three Types of Music During Exercise on Heart Rate, Blood Pressure, Rating of Perceived Exertion and Fatigue Onset Time | **WI** |
| Musical Information Increases Physical Performance for Synchronous but not Asynchronous Running | **WI** |
| The Impact of Cell Phone Use on the Intensity and Liking of a Bout of Treadmill Exercise | **WO** |
| Performance Enhancement With Music in Rowing Sprint | **WP** |
| The Effects of T’ai Chi with Asynchronous Music on the Health of Older Women: A Pilot Study | **WP** |
| The Effects of a Program Combining Exercise and Music on Promoting Exercise Continuance and Psychological Factors in Older People | **WP** |
| On the Role of Lyrics in the Music–Exercise Performance Relationship | **WI** |
| The Effect of Vocal and Instrumental Music on Cardio Respiratory Variables, Energy Expenditure and Exertion Levels During Sub Maximal Treadmill Exercise | **WI** |
| Effects of Music on Exercise Performance | **WI** |
| The Effects of Listening to Music or Viewing Television on Human Gait | **WO** |
| An Investigation of Stride Interval Stationarity While Listening to Music or Viewing Television | **WO** |
| Aerobic Training with Rhythmic Functional Movement: Influence on Cardiopulmonary Function, Functional Movement and Quality of Life in the Elderly Women | **WP** |
| The Effects of Synchronous Music on 400-m Sprint Performance | **WP** |
| Effects of Exergame and Music on Acute Exercise Responses to Graded Treadmill Running | **WI** |
| Let’s Go: Psychological, Psychophysical, and Physiological Effects of Music During Sprint Interval Exercise | **WO** |
| The Effects of Slow- and Fast-Rhythm Classical Music on Progressive Cycling to Voluntary Physical Exhaustion | **WI** |
| Effect of Music on Perceived Exertion, Plasma Lactate, Norepinephrine and Cardiovascular Hemodynamics During Treadmill Running | **WI** |
| Self-Selected Music-Induced Reduction of Perceived Exertion During Moderate-Intensity Exercise Does Not Interfere with Post-Exercise Improvements in Inhibitory Control | **WO** |
| Effects of Bone-Conducted Music on Swimming Performance | **WP** |
| The Effect of Music Type on Running Perseverance and Coping with Effort Sensations | **WI** |
| Effects of Synchronous Music on Treadmill Running among Elite Triathletes | **WP** |
| Effect of Music Tempo on Exercise Performance and Heart Rate among Young Adults | **WI** |
| Effect of Different Types of Music on Exercise Performance in Normal Individuals | **WI** |
| Effect of Music and Dialogue on Perception of Exertion, Enjoyment, and Metabolic Responses During Exercise | **WO** |
| Effects of Music Tempo upon Submaximal Cycling Performance | **WI** |
| Comparison of Passive Sensory Stimulations on RPE During Moderate Intensity Exercise | **WI** |
| Effects of Music During Exercise on RPE, Heart Rate and the Autonomic Nervous System | **WI** |
| Effect of Background Music on Maximum Acceptable Weight of Manual Lifting Tasks | **WI** |
| EFFECTS OF SELF-SELECTED MUSIC ON MAXIMAL BENCH PRESS STRENGTH AND STRENGTH ENDURANCE | **WO** |
| Physiological and Psychophysical Responses to Listening to Music during Warm-Up and Circuit-Type Resistance Exercise in Strength Trained Men | **WO** |
