# Supplementary material for: Music during anaerobic exercise in physically active adults: task-dependent evidence for repetition performance and affective valence, with uncertain maximal-performance effects
Source: Front Sports Act Living. 2026 Jul 8;8:1849596. doi: 10.3389/fspor.2026.1849596 (PMC13388559; doi:10.3389/fspor.2026.1849596)
Supplement: Supplementary file 3 [file Table3.docx]

**PubMed:** (music[Title/Abstract]) AND (((sport[Title/Abstract]) OR (exercise[Title/Abstract])) OR (“physical activity”[Title/Abstract])) Filters:English Sort by:Most Recent

**Web of science:** (TS=(music AND (sport OR exercise OR “physical activity”))) AND Language:(English)

**ScienceDirect:**Title, abstract, keywords:music AND (sport OR exercise OR "physical activity")

**Scopus:**Title, abstract, keywords:music AND ( sport OR exercise OR "physical activity" )

**EBSCO host:**SU music AND SU (sport OR excerise OR "physical activity")
